# The Synergistic Use of IL-15 and IL-21 for the Generation of NK Cells From CD3/CD19-Depleted Grafts Improves Their *ex vivo* Expansion and Cytotoxic Potential Against Neuroblastoma: Perspective for Optimized Immunotherapy Post Haploidentical Stem Cell Transplantation

**DOI:** 10.3389/fimmu.2019.02816

**Published:** 2019-12-03

**Authors:** Annekathrin Heinze, Beatrice Grebe, Melanie Bremm, Sabine Huenecke, Tasleem Ah. Munir, Lea Graafen, Jochen T. Frueh, Michael Merker, Eva Rettinger, Jan Soerensen, Thomas Klingebiel, Peter Bader, Evelyn Ullrich, Claudia Cappel

**Affiliations:** ^1^Experimental Immunology, Department for Children and Adolescents, University Hospital Frankfurt, Goethe University, Frankfurt am Main, Germany; ^2^Division for Stem Cell Transplantation, Immunology and Intensive Care Medicine, Department for Children and Adolescents, University Hospital Frankfurt, Goethe University, Frankfurt am Main, Germany; ^3^Department for Children and Adolescents, University Hospital Frankfurt, Goethe University, Frankfurt am Main, Germany; ^4^German Cancer Consortium (DKTK), Partner Site Frankfurt am Main, Frankfurt am Main, Germany

**Keywords:** immunotherapy, NK cells, CD3/CD19 depletion, CIK cells, IL-21, IL-15, *ex vivo* expansion, neuroblastoma

## Abstract

Neuroblastoma (NB) is the most common solid extracranial tumor in childhood. Despite therapeutic progress, prognosis in high-risk NB is poor and innovative therapies are urgently needed. Therefore, we addressed the potential cytotoxic capacity of interleukin (IL)-activated natural killer (NK) cells compared to cytokine-induced killer (CIK) cells for the treatment of NB. NK cells were isolated from peripheral blood mononuclear cells (PBMCs) by indirect CD56-enrichment or CD3/CD19-depletion and expanded with different cytokine combinations, such as IL-2, IL-15, and/or IL-21 under feeder-cell free conditions. CIK cells were generated from PBMCs by *ex vivo* stimulation with interferon-γ, IL-2, OKT-3, and IL-15. Comparative analysis of expansion rate, purity, phenotype and cytotoxicity was performed. CD56-enriched NK cells showed a median expansion rate of 4.3-fold with up to 99% NK cell content. The cell product after CD3/CD19-depletion consisted of a median 43.5% NK cells that expanded significantly faster reaching also 99% of NK cell purity. After 10–12 days of expansion, both NK cell preparations showed a significantly higher median cytotoxic capacity against NB cells relative to CIK cells. Remarkably, these NK cells were also capable of efficiently killing NB spheroidal 3D culture in long-term cytotoxicity assays. Further optimization using a novel NK cell culture medium and a prolonged culturing procedure after CD3/CD19-depletion for up to 15 days enhanced the expansion rate up to 24.4-fold by maintaining the cytotoxic potential. Addition of an IL-21 boost prior to harvesting significantly increased the cytotoxicity. The final cell product consisted for the major part of CD16^−^, NCR-expressing, poly-functional NK cells with regard to cytokine production, CD107a degranulation and antitumor capacity. In summary, our study revealed that NK cells have a significantly higher cytotoxic potential to combat NB than CIK cell products, especially following the synergistic use of IL-15 and IL-21 for NK cell activation. Therefore, the use of IL-15+IL-21 expanded NK cells generated from CD3/CD19-depleted apheresis products seems to be highly promising as an immunotherapy in combination with haploidentical stem cell transplantation (SCT) for high-risk NB patients.

## Introduction

Neuroblastoma (NB) is the most common extracranial solid tumor in childhood and causes 15% of cancer-related mortality in children. The majority of cases are diagnosed before the age of 5 years, and 30% of cases are diagnosed within the first year of life. Around half of the patients are currently classified as high-risk for disease relapse, with a 5-year event-free survival (EFS) of <40% despite intensive multimodal therapy (1–3). Current therapeutic approaches for high-risk NB include surgery, radiotherapy [iodine (I-131) Metaiodobenzylguanidine (MIBG) therapy or external beam radiation] and myeloablative chemotherapy, followed by autologous stem cell rescue. Furthermore, immunotherapies using monoclonal antibodies against NB cell membrane antigens such as anti-GD2 (e.g., Dinutuximab ch14.18/SP2/0; Dinutuximab-beta ch14.18/CHO) have gained increasing clinical significance (4, 5).

In addition, for children with relapsed or refractory high-risk NB, hematopoietic stem cell transplantation (SCT) has been shown to be a feasible and promising treatment that can induce long-term remission in some patients with tolerable side effects (6–8). In this context, haploidentical SCT from mismatched family donors is an important therapeutic option for patients lacking a human leukocyte antigen (HLA)-matched donor. The removal of potentially alloreactive T cells from the graft by CD3/CD19-depletion allows HLA barriers to be overcome and reduces the induction of harmful graft-versus-host-disease (GvHD). While the risk of EBV post-transplant lymphoproliferative disease (PTLD) is reduced by depletion of CD19^+^ B cells, CD3/CD19-depleted grafts also seem to facilitate engraftment and to support the graft-versus-leukemia/tumor (GvL/T) effect (9).

The beneficial GvL/T effect is predominantly mediated by NK cells that are key players of the innate immune system and potent effector cells participating in the defense of viral-infected and malignant cells (10). NK cells compromise ~6–12% of lymphocytes in the peripheral blood (PB) and are characterized by surface expression of CD56 and the lack of T cell antigens such as CD3 or T cell receptors (CD56^+^CD3^−^). Human NK cell subsets in the PB can further be subdivided into a major CD56^dim^CD16^+^ highly cytotoxic population, expressing the Fcγ receptor III CD16 and therefore exerting antibody-dependent cell-mediated cytotoxicity (ADCC), and a minor CD56^bright^CD16^−^ immune regulatory population with a potent cytokine producing capacity (11).

NK cell cytotoxicity is mediated by a balanced system processing signals from activating and inhibitory receptors (12, 13). Activating receptors, such as natural cytotoxicity receptors (NCRs) and the NK group 2D (NKG2D) receptor can be triggered by an enhanced surface expression of stress-induced ligands on abnormal cells (14, 15). Inhibitory signals are mainly mediated by killer immunoglobulin-like receptors (KIR) that recognize major histocompatibility complex (MHC) class I molecules, which are highly present on endogenous healthy cells. Most transformed and virally infected cells down-regulate their MHC class I surface expression (“missing-self”) to evade cytotoxic T cell recognition, which renders them sensitive to NK cell killing (16, 17).

Based on the donor cell mediated GvL/T effect, adoptive post-transplantation immunotherapeutic strategies using donor-derived immune cells have been established. To further increase the anti-tumor efficacy, donor cells can be *ex vivo* expanded and stimulated by different cytokines, generating highly activated NK or cytokine-induced killer (CIK) cell products. A synergistic effect of haploidentical SCT and cell-based immunotherapy seems probable, but few clinical results from the treatment of high-risk NB have yet been published (18–20).

Manufacturing sufficient effector cell doses for repetitive cell applications to overcome suppressive tumor escape mechanisms still remains a challenge. Therefore, we analyzed the expansion capacity as well as the cytotoxic potential upon different cytokines of either CD56-enriched or CD3/CD19-depleted NK cells compared to CIK cells against human NB cell lines *in vitro*.

## Materials and Methods

### Generation of NK Cells and CIK Cells

**Figure 1:**
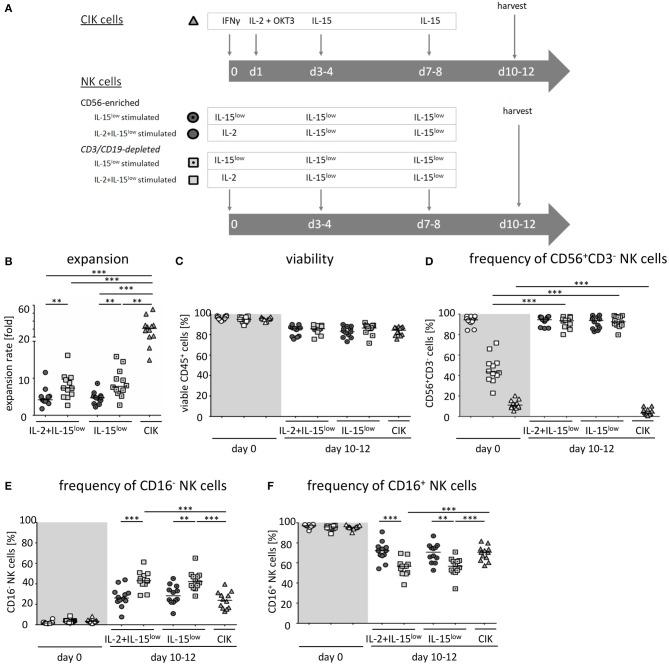
*Ex vivo* expansion and characterization of CIK and NK cells. **(A)** CIK cells were expanded by *ex vivo* stimulation with IFNγ, IL-2, OKT3, and IL-15 over a period of 10–12 days (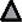). NK cells were purified by either CD56-enrichment (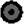

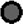
) or CD3/CD19-depletion (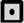

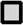
) and *ex vivo* stimulated with IL-2+IL-15 (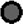

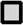
) or solely IL-15 (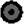

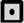
) for 10–12 days. **(B)** CD56-enriched NK cells expanded 4.3-fold independent of the use of IL-15 alone or in combination with IL-2 (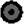

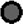
), NK cells in CD3/CD19-depleted cell products expanded significantly higher under both cytokine stimulation conditions (median 7.5-fold) (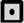

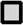
). CIK cells showed highest expansion rate of 30.8-fold, referring to the total cell composite of NK, T and NK-like T cells (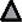) **(C)** All three cell products showed a high viability of >95% following purification procedure on day 0 (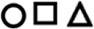) and remained at >85% during expansion procedure independent of the cytokine additive. **(D)** CD56-enriched cells on day 0 contained a median 94.5% NK cells, CD3/CD19-depleted cells products contained a median 43.5% NK cells, and CIK cells a median 11.5%. Upon cytokine stimulation for 10–12 days, NK cell purity remained high (94.6 and 93.8%) in CD56-enriched, and significantly increased in CD3/CD19-depleted cell products (93.6 and 92.5%) under IL-2+IL-15 or IL-15 stimulation, respectively. In contrast, the frequency of NK cells decreased to only 3.3% during CIK cell expansion. **(E)** The frequency of the CD16^−^ NK cell subpopulation significantly increased during IL-2+IL-15 or IL-15 cytokine stimulation, especially within CD3/CD19-depleted NK cell products (CD56-enriched 26.1 and 28.3%; CD3/CD19-depleted 43.0 and 42.3%), while the CD16^+^ NK cell subpopulation correspondingly decreased **(F)** (*n* = 12 independent experiments, median fold expansion rate day 10–12 compared to day 0, gated on viable cells (DAPI negative): CD56^+^CD3^−^NK cells **(B)**, CD45^+^ cells **(C)**, CD56^+^CD3^−^NK cells **(D)**, CD16^−^ NK cells **(E)**, CD16^+^ NK cells **(F)**. Differences were considered significant for *p* < 0.01 (**) and *p* < 0.001 (***).

**Figure 2:**
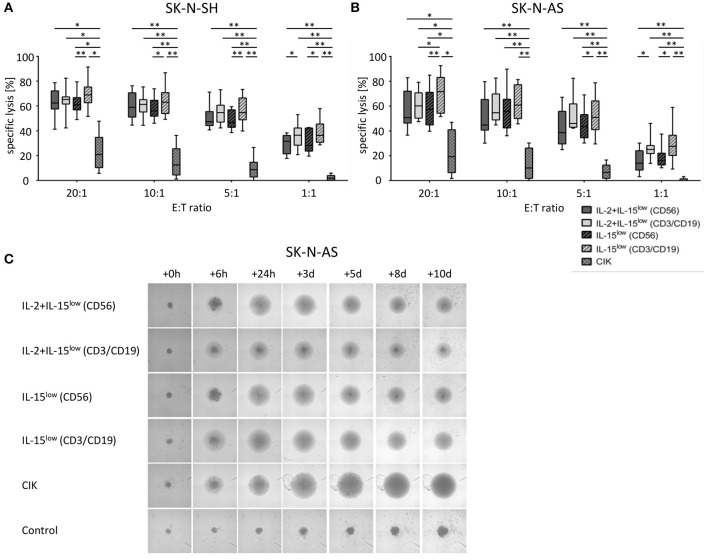
Cytotoxic potential and long-term cytotoxicity of CIK and NK cells against NB target cells. Specific lysis of the NB cell lines SK-N-SH **(A)** and SK-N-AS **(B)** was evaluated by Europium release assay. Both NB cell lines were efficiently lysed by either CD56-enriched (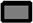

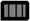
) or CD3/CD19-depleted (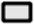

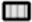
) NK cells; while CD3/CD19-depleted NK cells (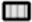) killed significantly better relative to CD56-selected (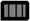) NK cells at all E:T ratios. In addition, there was an upward tendency for IL-15 to be superior to IL-2+IL-15 treatment, which was statistically relevant for CD3/CD19-depleted NK cells vs. SK-N-AS cells at the 20:1 E:T ratio, only. CIK cell-mediated cytotoxicity was significantly lower with a median cell lysis of 12.5% for SK-N-SH and 10.1% for SK-N-AS cells (all E:T ratio 10:1). Effector to target (E:T) ratios 20:1, 10:1, 5:1 and 1:1, *n* = 8–9 independent results, experiments performed in triplicate, incubation time: 3 h, box-and-whisker plots show median, 25th−75th percentiles, Min-Max. **(C)** Tumor spheroids were produced from 10,000 SK-N-AS cells and co-incubated with 200,000 CIK or NK cells. As a control the dynamics of tumor spheroids without effector cells were observed. The cultures were imaged via a Celigo cell cytometer after 6 h, 24 h, 3, 5, 8 and up to 10 days. All NK cells sustained tumor growth but only IL-15 stimulated NK cells generated from CD3/CD19 depleted PBMCs were able to completely eradicate tumor cells. Also CIK cells infiltrated and lysed NB tumor spheroids (*n* = 1 representative of 3 independent experiments). Differences were considered significant for *p* < 0.05 (*), *p* < 0.01 (**).

NK cells and CIK cells were generated from PBMCs from buffy coats of healthy donors by density gradient centrifugation. In experiments directly comparing NK and CIK cell proliferation and cytotoxicity (Figures 1, 2) the NK cells and CIK cells were generated from PBMCs isolated from buffy coats of the same donor. Donors gave their written informed consent and the use of the buffy coats was approved by the medical ethics committee of the University Hospital Frankfurt (approval no. 329/10). CIK cells were generated and cultured as described previously (21). In short, PBMCs were adjusted to a concentration of 3 × 10^6^ cells/ml in X-VIVO^TM^10 medium (Lonza, Verviers, Belgium) supplemented with 5% heat-inactivated human fresh frozen plasma (FFP) (DRK-Blutspendedienst Baden-Württemberg-Hessen, Frankfurt/Main, Deutschland) and 1% penicillin/streptomycin (Invitrogen, Thermo Fisher, Waltham, MA, USA) in tissue culture-treated plates (Thermo Fisher, Waltham, MA, USA; Sarstedet, Nümbrecht, Germany; Corning NY, USA). On day 0 they received 1,000 U/ml IFN-γ (Imukin®, Boeringer Ingelheim Pharma, Germany). On day 1, 100 ng/ml OKT- (anti-CD3 antibody, MACS GMP CD3 pure, Miltenyi Biotec, Bergisch Gladbach, Germany) and 500 U/ml IL-2 (ProleukinS, Novartis Pharmaceuticals, Horsham, UK) were added. On days 3–4 and 7–8 the cell density was set to a concentration of 1 × 10^6^ cells/ml and cells were treated with 50 ng/ml IL-15 (Peprotech, Rocky Hill, CT, USA). CIK cells were harvested on days 10–12 (Figure 1A).

**Figure 3:**
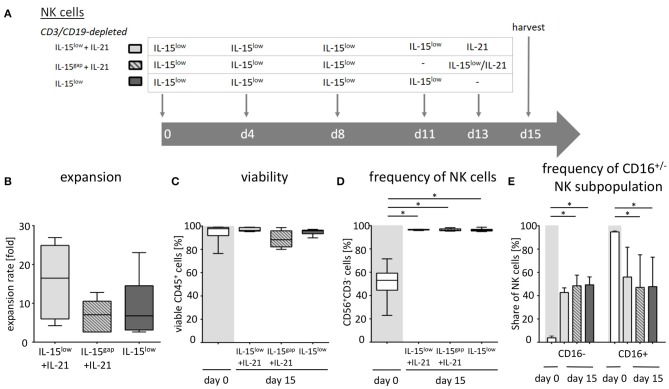
*Ex vivo* expansion and characterization of IL-15 and IL-15+IL-21 stimulated NK cells following CD3/CD19-depletion. **(A)** NK cells were purified by CD3/CD19-depletion and *ex vivo* stimulated with IL-15 (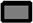) or with a combination of IL-15 and IL-21 (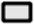

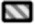
) for 15 days. On day 11, cells were either treated with IL-15 (IL-15^low^
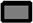

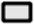
) or the supernatant was removed and no cytokines were added (IL-15^gap^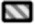). IL-21 was added as a cytokine boost 48 h prior to harvest. **(B)** NK cells in the IL-15^low^ protocol 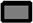) expanded 6.8-fold. An IL-21 boost was able to further enhance proliferation, irrespective of gap or continuous treatment. Expansion rates reached 7.1-fold in the IL-15^gap^+ IL-21 protocol (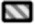) and 16.5-fold in the IL-15^low^+ IL-21 protocol (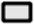) (statistically not significant differences). **(C)** All cell products showed a high viability of median 97.5% following the purification procedure on day 0 (white symbols gray background) and remained >80% during the expansion procedure independent of the cytokine additive. However, the gap treatment led to the lowest viability (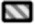). **(D)** Purified CD3/CD19-depleted cells on day 0 contained a median 53.1% NK cells. Upon cytokine stimulation for 15 days, NK cell purity significantly increased in CD3/CD19-depleted cell products regardless of the cytokine combination. **(E)** The frequency of the CD16^−^ NK cell subpopulation significantly increased during *ex vivo* stimulation within all protocols (*n* = 5–6 independent experiments, **(B)** median fold expansion rate on day 15 compared to day 0, **(C)** gated on viable 7-AAD^−^ CD45^+^ cells, **(D)** CD56^+^CD3^−^ NK cells, **(E)** CD16^−^ NK cells. Box-and-whisker plots show median, 25th−75th percentiles, Min-Max. Bar graphs show median and interquartile range. Differences were considered significant for *p* < 0.05 (*).

NK cells were isolated from PBMCs using either the EasySep® Human NK cell enrichment kit or EasySep® Human CD3 and CD19 positive selection kits (STEMCELL Technology, Vancouver, Canada). Cells were adjusted to a concentration of 3 × 10^6^ cells/ml in either X-VIVO^TM^10 medium or NK MACS® medium supplemented with 1% NK MACS supplements (Miltenyi Biotec, Bergisch Gladbach, Germany) and 5% human plasma (HP) type AB and 1% penicillin/streptomycin in 48-well cell culture plates (Thermo Fisher Scientific, Roskilde, Denmark). Analogous to CIK cells, NK cell density was adjusted to 1 × 10^6^ cells/ml on subsequent culture days. In detail, we suspended, harvested and counted cells, took out needed cells, diluted to 1 × 10^6^/ml with fresh medium and added cytokine in the corresponding concentration. To optimize *ex vivo* cultivation of NK cells, different stimulation protocols were tested. In the IL-15^low^ protocol, NK cells were stimulated with 10 ng/ml IL-15 every 3–4 days for a period of either 10–12 or 15 days (Figures 1A, 3A, 5A). In the IL-2+IL-15^low^ protocol NK cells received 500 U/ml IL-2 on day 0 and 10 ng/ml IL-15 every 3–4 days until days 10–12 (Figure 1A). For the IL-15^low^+IL-21 protocol NK cells were cultivated with 10 ng/ml IL-15 every 3–4 days including day 11. On day 13, 25 ng/ml IL-21 (Peprotech Rocky Hill, CT, USA) was added, and cells were harvested on day 15 (Figures 3A, 5A). In the IL-15^gap^+IL-21 protocol, cells were cultured for 11 days with addition of 10 ng/ml IL-15 every 3–4 days and then collected and centrifuged. The supernatant was removed in order to abolish traces of cytokines. On day 13, 10 ng/ml IL-15 and 25 ng/ml IL-21 were added (Figure 3A) (22, 23).

### NB Cell Lines

The human NB cell lines SK-N-SH (ATCC® HTB-11™) and SK-N-AS (ATCC® CRL-2137™) were cultured in IMDM medium (Gibco, Thermo Fisher) supplemented with 10% fetal bovine serum (FBS) (Biochrom, Berlin, Germany) and 1% penicillin/ streptomycin (Invitrogen, Thermo Fisher, Waltham, MA, USA) at 37°C and 5% CO_2_. The cells were split once or twice a week using Accutase solution (Sigma-Aldrich, St. Louis, MO, USA) to detach adherent cells from the cell culture flask. MHC class I is expressed to a low extent on SK-N-SH and at higher levels on SK-N-AS cells (24, 25).

### Flow-Cytometric Analysis

Flow cytometric analysis was performed on a FACS Canto 10c and a FACS Celesta instrument (both BD Biosciences, San Jose, CA, USA). NK cells and CIK cells were analyzed for the expression of various surface molecules on day 0 and the day of harvest. Flow cytometric phenotyping was performed for the most promising protocols on the day of harvest. The following monoclonal antibodies were used:

AF488: NKp30 (#210845), NKG2C (#134591) (both R&D Systems Wiesbaden, Germany), APC: CD57 (NK-1, Biolegend, San Diego, CA, USA), CD178/FASL (NOK-1), NKG2D (ON72, Beckman Coulter, Krefeld, Germany), CD107a (H4A3, BioLegend), APC-A700: CD16 (3G8, Biolegend), CD56 (N901, Beckman Coulter), BB515: CD19 (HIB19), BUV395: CD3 (SK7), BUV737: CD20 (2H7), BV421: CD56 (NCAM16.2), Zombie Violet (BioLegend), BV510: CD45 (HI30), BV605: CD25 (2A3), CD69 (FN50), BV711: CD14 (M5E2, Biolegend), FITC: CD3 (UCHT1, Beckman Coulter), CD226 (DX11), KIR2D (NKVFS1, Miltenyi Biotec, Bergisch Gladbach, Germany), IFNy (B27), PC7: CD19 (HIB19, Biolegend), CD184/CXCR4 (12G5), PE: CD16 (3G8, Biolegend), NKG2A (Z199, Beckman Coulter), NKp44 (P44-8.1), NKp46 (9E2, Biolegend), PD1 (PD1.3.1.3, Miltenyi), TRAIL (RIK-2, Biolegend), CD56 (NCAM16.2), PE Vio770: 2B4 (REA112, Miltenyi), PerCP: CD3 (UCHT1, Biolegend), CD14 (TÜK4, Miltenyi), CD19 (HIB19, Biolegend), CD16 (3G8, BioLegend), V450: CD3 (UCHT1), CD14 (MøP9), CD19 (HIB19) All antibodies were purchased from BD Biosciences if not otherwise specified.

**Figure 4:**
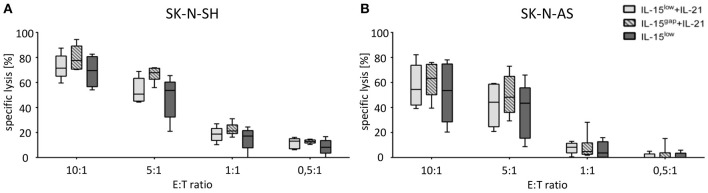
Cytotoxic potential of IL-15+IL-21 stimulated CD3/CD19-depleted NK cells against NB target cells. Specific lysis of the NB cell lines SK-N-SH **(A)** and SK-N-AS **(B)** was evaluated by Europium release assay. Both NB cell lines were efficiently lysed by CD3/CD19-depleted NK cells. Median target cell lysis of NK cells treated in the IL-15^low^ protocol (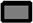) was 69. Five percent for SK-N-SH and 53.5% for SK-N-AS cells (E:T ratio 10:1). An additional IL-21 boost elevated cytotoxic activity of IL15-stimulated NK cells to a median cell lysis of 71.5% (IL-15^low^ + IL-21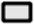) and 77.6% (IL-15^gap^ +IL-21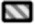) for SK-N-SH and 54.4% (IL-15^low^+IL-21) and 63.3% (IL-15^gap^ +IL-21) for SK-N-AS cells (all E:T ratio 10:1) (statistically not significant differences). Effector to target (E:T) ratios 10:1, 5:1, 1:1, and 0.5:1, *n* = 5–6 independent results, experiments performed in triplicate, incubation time: 3 h, box-and-whisker plots show median, 25th−75th percentiles, Min-Max.

**Figure 5:**
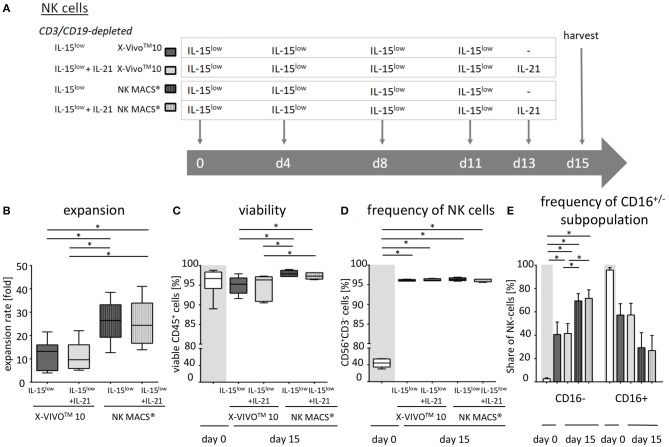
*Ex vivo* expansion and characterization of IL-15+IL-21 stimulated CD3/CD19-depleted NK cells cultured in optimized NK cell medium. **(A)** NK cells were purified by CD3/CD19-depletion and *ex vivo* stimulated with solely IL-15 (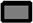

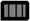
) or with a combination of IL-15 and IL-21 (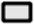

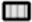
) for 15 days. IL-21 was added as a cytokine boost 2 days prior to harvest. NK cells were cultured in X-VIVO^TM^10 medium (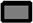

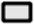
) or NK MACS® medium (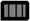

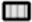
). **(B)** NK cells in X-VIVO^TM^10 medium expanded 13.2-fold (IL-15^low^) 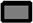 and 9.6-fold (IL-15^low^+ IL-21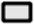). Expansion rates were significantly higher in NK MACS® medium at 26.4-fold (IL-15^low^ 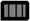) and 24.4-fold (IL-15^low^+IL-21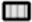). **(C)** All cell products showed a high viability with a median 96.5% following the purification procedure on day 0 (white symbols gray background) and remained >90% during the expansion procedure, independent of the cytokine additive. *Ex vivo* cultivation in NK MACS® medium even led to viability >96%. **(D)** Purified CD3/CD19-depleted cells on day 0 contained a median 54.4% NK cells. Upon cytokine stimulation for 15 days NK cell purity significantly increased in CD3/CD19-depleted cell products to >95% in all protocols. **(E)** The frequency of the CD16^−^ NK cell subpopulation significantly increased during *ex vivo* stimulation. In total, 42.7% (IL-15^low^ 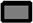) and 42.6% (IL-15^low^+ IL-21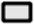) of NK cells were CD16^−^ after cultivation in X-VIVO^TM^10 medium. Percentages of CD16^−^ cells were significantly higher after cultivation in NK MACS® medium: 69.5% (IL-15^low^ 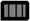) and 71.6% (IL-15^low^+ IL-21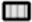) (*n* = 6 independent experiments, **(B)** median fold expansion rate day 15 compared to day 0, gated on: **(C)** viable 7-AAD^−^ CD45^+^ cells, **(D)** CD56^+^CD3^−^ NK cells, **(E)** CD16^−^ NK cells. Box-and-whisker plots show median, 25th−75th percentiles, Min-Max. Bar graphs show median and interquartile range. Differences were considered significant for *p* < 0.05 (*).

**Figure 6:**
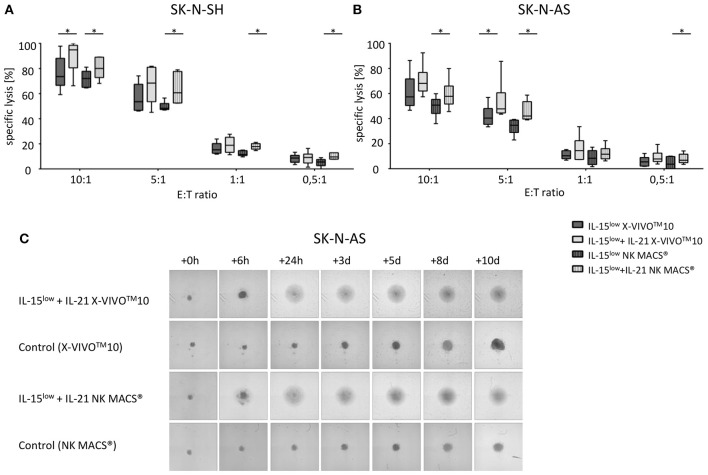
Cytotoxic potential and long-term cytotoxicity after optimization of NK cell cultivation. Specific lysis of the NB cell lines SK-N-SH **(A)** and SK-N-AS **(B)** was evaluated by Europium release assay. Both NB cell lines were efficiently lysed by CD3/CD19-depleted NK cells. Median target cell lysis of NK cells treated in the IL-15^low^ protocol in X-VIVO^TM^10 (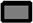) was 73.6% for SK-N-SH and 57.4% for SK-N-AS. An IL-21 boost (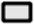) significantly elevated the cytotoxic activity of IL15-stimulated NK cells in X-VIVO^TM^10 medium to a median cell lysis of 94.92% for SK-N-SH and 68.09% for SK-N-AS cells. IL-21 also significantly increased target cell lysis in NK MACS® medium. Cultivation in NK MACS® medium resulted in slightly lower cytotoxic activity with median cell lysis of 70.3% (IL-15^low^ 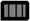) and 80.1% (IL-15^low^+ IL-21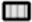) against SK-N-SH and 50.7 and 57.8% against SK-N-AS (all E:T ratio 10:1). E:T ratios 10:1, 5:1, 1:1, and 0.5:1, *n* = 6 independent results, experiments performed in triplicate, incubation time: 3 hours, box-and-whisker plots show median, 25th−75th percentiles, Min-Max. **(C)** Tumor spheroids were produced from 10,000 SK-N-AS cells and co-incubated with 200,000 NK cells. As a control the dynamics of tumor spheroids without effector cells were observed in both cell culture media. The cultures were imaged via a Celigo cell cytometer after 6 h, 24 h, 3, 5, 8 and up to 10 days. IL-15^low^+IL-21 stimulated NK cells grown in both cell culture media were able to completely eradicate tumor spheroids in this 10 day long-term cytotoxicity assay (*n* = 1 representative of 3 independent experiments). Differences were considered significant for *p* < 0.05 (*).

**Figure 7:**
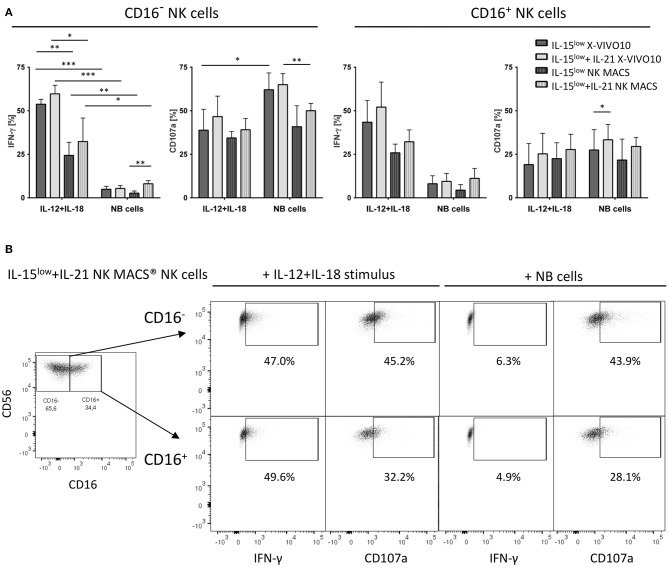
CD107a degranulation and IFN-γ production. **(A)** Intracellular IFN-γ expression and degranulation potential indicated by CD107a expression of cytokine stimulated NK cells cultured in X-VIVO™10 or NK MACS® media was assessed after 15 days of cultivation with the IL-15^low^ or IL-15^low^+IL-21 stimulation protocol. Cells were either co-incubated with SK-N-AS NB target cells (E:T ratio 1:1) or stimulated with IL-12+IL-18 mimicking stimulation by dendritic cells. After the total incubation time of 4 h, cells were stained and measured by flow cytometry. IFN-γ and CD107a expression was compared to unstimulated cells in each cultivation setting used as negative control. Both NK cell subsets produced IFN-γ upon cytokine stimulation and target cell co-incubation, with higher levels after the cytokine stimulus, which was statistically significant for the CD16^−^ subset. Similar effects were seen in both media, except CD16^−^ NK cells grown in X-VIVO™10 produced significantly more IFN-γ upon IL-12+IL-18 cytokine stimulation. Target cell co-incubation and cytokine stimulation led to a high CD107a expression in both NK cell subpopulations, especially within the CD16^−^ NK cell population. Only small differences were seen between both cell culture media. Throughout all experiments, the additional IL-21 boost during NK cell cultivation enhanced IFN-γ and CD107a expression, which was even statistically significant in *n* = 2 settings (light gray vs. dark gray bars). Summary data show mean and SEM percentage of CD107a^+^ and IFN-γ^+^ NK cells (*n* = 4 independent results). **(B)** FACS plots show IFN-γ and CD107a expression in both CD16^−^ and CD16^+^ NK cell subpopulations of IL-15^low^+IL-21 NK cells grown 15 days in NK MACS® media. This stimulation protocol led to an outgrowth of the CD16^−^ NK cell subpopulation resulting in an inverse CD16^−^/CD16^+^ distribution. The short-term stimulus of IL-12+IL-15 and target cell co-incubation, demonstrated that both NK cell subpopulations are capable of IFN-γ production and CD107a expression. Thereby CD107a expression was higher present on CD16^−^ NK cells, while IFN-γ was produced equally by both subsets. FACS plots gated on viable Zombie Violet^−^ CD3^−^CD56^+^ NK cells (density plots show one representative result from *n* = 4 independent experiments). Differences were considered significant for *p* < 0.05 (*), *p* < 0.01 (**), and *p* < 0.001 (***).

Depending on the panel either strongly diluted DAPI (Biolegend, San Diego, CA, USA) (experiments in Figures 1, 2) or 7-Actinomycin D (7-AAD) (BD Biosciences San Jose, CA, USA) (experiments in Figures 3–6) was used for the assessment of cell viability. Absolute lymphocyte subset counts were calculated via a dual platform using the flow cytometric data and cell counts acquired via a COULTER Ac.T diff Analyzer (Beckman Coulter, Krefeld, Germany). Flow-cytometric data was analyzed using FlowJo-software (Tree Star Inc., Ashland, OR, USA).

### Europium Release Assay

To test the cytotoxicity of CIK and NK cells against the human NB cell lines SK-N-SH and SK-N-AS, the non-radioactive europium release assay was used as described previously (26) and modified for adherent cell lines (27). In short, human NB cell lines (target cells) were labeled with BATDA (Perkin Elmer, Boston, MA, USA) under constant shaking (400 rpm) at 37°C, 5% CO_2_ to prevent cell adhesion. Cells were washed in medium supplemented with Probenecid (Santa Cruz, Dallas, Texas, USA), an inhibitor of multidrug-resistance associated proteins, to avoid spontaneous release of TDA into the supernatant. Target cells were co-cultured with NK cells or CIK cells (effector cells) for 3 h in triplicates at effector-to-target (E:T) ratios of 20:1, 10:1, 5:1, 1:1, and 0.5:1 at 37°C. Target cells were also cultured in medium without effector cells to determine spontaneous cell lysis. The maximum lysis was obtained by adding 16% Triton-X solution (AppliChem, Darmstadt, Germany) to target cells. After co-incubation, 20 μl of supernatant was taken from each well and incubated for 15 min in the dark and under shaking (250 rpm) with 200 μl of europium solution (Perkin Elmer, Boston, MA, USA). The fluorescence signal, which directly correlates to tumor cell lysis, was measured by a multilabel plate reader (Victor 1420 multilabel counter, Perkin Elmer, Boston, MA, USA). Percentage of lysis was calculated as follows: specific lysis = [(lysis – spontaneous lysis)/(maximal lysis – spontaneous lysis)]^*^100.

### Co-culture in the Presence of Tumor Spheroids

Three-dimensional (3D) tumor spheroids were obtained by placing 10,000 SK-N-AS cells into one well each of an ultra-low attachment (ULA) 96-well round-bottom plate without additional coating (Corning Incorporated, Corning NY, USA). Culture plates were centrifuged at 1,000 g for 10 min. Every 3 to 4 days, half of the medium was replaced and on day 4 200,000 CIK or NK cells were added per well. The dynamics of the tumor spheroid were automatically imaged using a Celigo cell cytometer (Nexcelom Bioscience, Lawrence, MA, USA) with the colony counting embryoid body application at time points of 0, 6, 24 h and subsequently every 24 h for a total of 10 days (28, 29).

### CD107a Degranulation Assay and IFN-γ Analysis

Intracellular IFN-γ expression and degranulation potential of cytokine stimulated NK cells cultured in X-VIVO™10 or NK MACS® media was assessed with cells harvested on day 15 of cultivation. Cells were incubated in 96-well-U-bottom-plates for a total time of 4 h, either co-incubated with SK-N-AS target cells (E:T ratio 1:1) or stimulated with cytokines at 37°C and 5% CO_2_. SK-N-AS target cell stimulation started from the beginning of co-incubation. After 1 h, cells were incubated with anti-human CD107a, followed by additional 2 h incubation with Monensin/ GolgiStop™ (BD Biosciences). Simultaneously, IL-12 (Peprotech, 96 ng/ml) and IL-18 (MBC, 98.7 ng/ml) were added to the wells not stimulated so far. After the total incubation time of 4 h, cells were washed, blocked with 1 μg of human IgG and stained with Zombie Violet™ Fixable Viability Kit (Biolegend, 5 μl, pre-diluted 1:10 in DPBS) for live/death discrimination. Post washing, cells were stained for 20 min with CD45, CD56, CD16, CD3, CD14, and CD19 in brilliant stain buffer. After a further washing step, they were fixed with formaldehyde solution (3.7% final concentration) by a 15 min incubation. In the end, permeabilization and intracellular staining took place by incubating cells with saponin buffer (0.2% saponin, 1% bovine serum albumin, Sigma Aldrich) and anti-human IFN-γ for further 30 min. For final analysis, cells were washed with perm/wash buffer solution (BD Biosciences, prediluted 1:10 with distilled water), and measured by flow cytometry (Canto 10C).

### Statistical Analysis

Results were statistically analyzed using GraphPad Prism 6 (GraphPad Software, San Diego, CA, USA). The data were compared by a Wilcoxon matched paired signed rank test. Differences were considered significant for *p* < 0.05 (^*^), 0.01 (^**^), and 0.001 (^***^).

## Results

### *Ex vivo* Expansion and Characterization of CIK and NK Cells

Aiming to manufacture sufficient effector cell doses and to evaluate the most promising cell population to target NB, we compared different cell purification (CD56-enriched vs. CD3/CD19-depleted NK cell) and cytokine stimulation procedures (IL-15 vs. IL-2+IL-15) (Figure 1A). For better comparison, NK cell stimulation was adjusted to the well-established stimulation protocol for GMP-grade CIK cell production (30).

Highly purified CD56-enriched NK cells showed a median expansion rate of 4.3-fold (range 1.8–11.6) after 10–12 days, independent of the use of IL-15 alone or in combination with an initial IL-2 boost at day 0 (Figure 1B, Table 1). In contrast, NK cells within CD3/CD19-depleted cell products expanded significantly higher with both cytokine stimulation settings (median 7.5-fold, range 2.7–16.2) (Figure 1B). CIK cells showed the most prominent expansion rate of 30.8-fold, referring to the total cell composite of NK, T and NK-like T cells (Figure 1B).

**Table 1:** Summary statistics to experiments shown in Figure 1.

**Purification method**		**CD56-enriched**	**CD3/CD19-depl**.	**CD56-enriched**	**CD3/CD19-depl**.
**NK cell stimulation**		**IL-2+IL-15**		**IL-15**	
NK cell expansion rate	[x-fold]	4.2 (1.8–11.6)	7.4 (2.7–16.2)	4.7 (2.3–8.6)	7.8 (2.7–15.9)
Viability	[%] of WBC	86.3 (75.5–89.9)	85.7 (75.5–89.7)	83.1 (73.1–89.8)	86.4 (71.6–91.8)
Purity NK cells	[%] of WBC	94.6 (86.1–97.8)	93.6 (80–97.7)	93.8 (82.9–99)	92.5 (79.8–98.6)
CD16^−^ NK cells	[%] of NK	26.1 (7.6–43.9)	43.0 (28.7–61.3)	28.3 (10.9–45.2)	42.3 (28.0–65.1)
CD16^+^ NK cells	[%] of NK	72.2 (54.2–91)	56.7 (38.3–69.5)	70.7 (52.7–86.9)	56.8 (34.3–70.9)
Ratio CD16^−^/CD16^+^		0.4	0.7	0.4	0.8
T cell contamination	[%] of WBC	1.0 (0.07–5)	0.03 (0–8.4)	0.34 (0.05–2.5)	0.05 (0–1.5)
NKT cell contamination	[%] of WBC	0.52 (0.05–3.5)	0.07 (0–1.1)	0.16 (0.02–1.2)	0.03 (0–1.2)
B cell contamination	[%] of WBC	0.04 (0–4)	0.12 (0.01–3)	0.08 (0–4)	0.09 (0.04–2.4)

*CD56^+^CD3^−^ NK cells were analyzed on day of harvest (day 10–12). Median (range) of expansion rate, purity, viability, NK cell subpopulations and ratios, as well as T, NK-like T and B cell contamination was assessed by flow cytometric analyses. NK, natural killer; NKT, NK-like T; IL, interleukin; WBC, white blood cell; CD3/CD19-depl., CD3/CD19-depleted*.

All three cell products showed a high viability of >95% following the purification procedure on day 0 (Figure 1C, Table 1). Independent of the cytokine additive, median viability of CD56-selected as well as CD3/CD19-depleted NK and CIK cells remained >85% during expansion (Figure 1C).

Composition of the different cell products was evaluated regarding the frequency of CD3^−^CD56^+^ NK cells (Figure 1D, Table 1) (incl. immune regulatory CD56^bright^CD16^−^ (Figure 1E, Table 1) and cytotoxic CD56^dim^CD16^+^ subpopulations (Figure 1F, Table 1), CD3^+^ T cells (Supplementary Figure 1A), CD3^+^CD56^+^ NK-like T cells (Supplementary Figure 1B), and CD19^+^ B cells (Figure 1C). Composition of the cell products used for expansion differed distinctly depending on the purification procedure. CD56-enriched cell products mainly consisted of highly purified NK cells of in median 94.5%, with minimal overall T, NK-like T and B cell contamination of 0.02%. CD3/CD19-depleted cells products consisted of in median 43.5% NK cells as well as minimal overall T and NK-like T contamination of 0.02%, but with a slightly higher B cell amount. Notably, CD3/CD19-depleted cell products additionally compromised a median of 33.9% (range 16.4–51.8%) monocytes. Thereby, monocytes analyzed in CD3/CD19-depleted cell products highly expressed the IL-15 receptor alpha (IL-15Rα, CD215) on the surface, and expression even further increased early in co-culture (*n* = 3, data not shown). Isolated PBMCs serving as starting material for CIK cell generation contained 11.5% NK cells, 54.7% T cells, 3.7% NK-like T cells and 4.4% B cells.

Upon IL-2+IL-15 and IL-15 cytokine stimulation, CD56-enriched NK cell products contained >94% NK cells, <1.0% T cells, <0.52% NK-like T cell and <0.08% B cells. Thereby, T and NK-like T cell contamination was significantly higher in CD56-enriched NK cell products and if IL-2 was added. CD3/CD19-depleted NK cell products reached >92.5% NK cell purity also without relevant T, NK-like T and B cell contamination. Again, T cell contamination was higher if IL-2 was added, although not statistically relevant (Figure 1, Supplementary Figure 1, and Table 1).

Following expansion, CIK cells contained 3.3% NK cells (range 0.6–10.1%), 84.2% T cells (range 69.6–97.4), 6.2% NK-like T cells (range 1.2–20.7), and 0.6% B cells (range 0–0.7%). Thereby, the content of T and NK-like T cell increased while the NK cell count decreased.

Overall, IL-15 stimulated CD3/CD19-depleted NK cell products revealed the lowest T and NK-like T cell contamination of all NK cell products. In addition, the CD16^−^ immune regulatory NK cell subpopulation significantly increased during cytokine stimulation, especially within the CD3/CD19-depleted NK cell products. As shown in Table 1, the ratio of CD16^−^/CD16^+^ NK cells on the day of harvest was the lowest, when NK cells were purified by CD56 enrichment leading to a NK cell product consisting of predominantly CD16^+^ NK cells (ratio 0.4). NK cells expanded from CD3/CD19 depleted cell products led to a higher proportion of CD16^−^ NK cells in the final cell product (ratio 0.7 for IL-2+IL-15 and 0.8 for IL-15 stimulation) independent of the cytokine combinations.

### Cytotoxic Potential of CIK and NK Cells Against NB Target Cells

Using the Europium release assay, the NK or CIK cell-mediated cytotoxicity against two different NB cell lines, SK-N-SH (Figure 2A) and SK-N- AS (Figure 2B), was evaluated. Both NB cells lines were efficiently lysed by either CD56-enriched or CD3/CD19-depleted NK cells with escalating E:T ratios. Thereby, NK cells isolated by CD3/CD19-depletion killed significantly better relative to CD56-selected NK cells in all E:T ratios. The median specific lysis of SK-N-SH cells by IL-15^low^ treated NK cells was 62.4%, 58.9%, 47.3% and 31.6% for CD56-enriched NK cells and 68.9, 63.0, 54.6, and 36.3% for CD3/CD19-depleted NK cells at E:T ratios of 20:1, 10:1, 5:1 and 1:1, respectively. In addition, there was a clear tendency for IL-15 to be superior to IL-2+IL-15 treatment. Notably, NK cells expanded after CD3/CD19-depletion with IL-15^low^ revealed the highest cytotoxic potential against both target cell lines. In contrast, CIK cell-mediated cytotoxicity was significantly lower in all E:T ratios; e.g., 12.5% (range 1.2–36.3%) and 10.1% (range 0.1–30.3%) CIK cells vs. SK-N-SH and SK-N-AS at the 10:1 ratio. Of note, CIK cell killing correlated with the amount of NK cells present in the individual heterogeneous CIK cell composite (data not shown).

To further address long-term cytotoxicity and mimic tumor lysis in a 3D setting, we used a tumor spheroid model. NB tumor spheroids built of SK-N-AS cells were co-incubated with CIK or NK cells following different stimulation procedures (Figure 2C). All NK cells were able to quickly infiltrate and lyse the tumor spheroids. However, only co-incubation with IL-15-stimulated NK cells generated from CD3/CD19-depleted PBMCs led to a complete lysis with no reoccurrence of target cells. CIK cells also infiltrated tumor spheroids and lysed target cells resulting in complete lysis of the tumor spheroids. In the total observation period of 10 days no tumor regrowth could be seen as well.

### Optimizing *ex vivo* NK Cell Activation Protocol for CD3/CD19-Depleted Cells by IL-21

As NK cells expanded with IL-15 after CD3/CD19-depletion showed the highest expansion rate and cytotoxic capacity against NB cell lines, we further aimed to optimize this protocol based on previous data indicating a further gain in NK cell cytotoxicity by a boost with IL-21 (23). Therefore, the cytokine stimulation has been prolonged by the addition of IL-21 prior to harvesting. In addition, a break of cytokine exposure prior to the IL-21 boost has been tested as this short “gap” in cytokine addition has been reported to improve NK cell expansion (22) (Figure 3A, Table 2). *Ex vivo* expansion with IL-15^low^ for 15 days led to a median expansion rate of 6.8-fold (range 2.6–23.1) (Figure 3B, Table 2). An additional boost with IL-21 on day 13 of culture was able to further enhance proliferation up to a median 16.5-fold (range 4.2–26.9), but not to a statistically relevant extent. If cells received only medium and no cytokines at day 11 prior to the IL-21 boost on day 13 (IL-15^gap^+IL-21), NK cells expanded 7.1-fold (range 2.6–12.8), only.

**Table 2:** Summary statistics to experiments shown in Figure 3.

**Purification method**		**CD3/CD19-depl**.		
**NK cell stimulation**		**IL-15+IL-21**	**IL-15+IL-21^**gap**^**	**IL-15**
NK cell expansion rate	[x-fold]	16.5 (4.2–26.9)	7.1 (2.6–12.8)	6.8 (2.6–23.1)
Viability	[%] of WBC	96.2 (95.2–99.1)	88.3 (80–98.7	96.0 (89.9–97.2)
Purity NK cells	[%] of WBC	96.7 (95.8–96.9)	96.0 (95.3–98.3)	96.2 (94.9–98.7)
CD16^−^ NK cells	[%] of NK	42.7 (16.4–46.7)	48.5 (19.2–57.6)	49.4 (18.1–56.1)
CD16^+^ NK cells	[%] of NK	56.0 (51.6–81.6)	47.1 (39.7–75.1)	47.8 (40.2–73)
Ratio CD16^−^/CD16^+^		0.7	1.0	1.0
T cell contamination	[%] of WBC	0.0 (0–0.01)	0.02 (0–0.04)	0.005 (0–0.37)
NKT cell contamination	[%] of WBC	0.19 (0.06–0.3)	0.24 (0.09–0.44)	0.16 (0.46–0.38)
B cell contamination	[%] of WBC	0.02 (0–0.03)	0.03 (0.01–0.11)	0.01 (0–0.18)

*CD56^+^CD3^−^ NK cells were analyzed on day of harvest (day 10–12). Median (range) expansion rate, purity, viability, NK cell subpopulations and ratios, as well as T, NK-like T and B cell contamination was assessed by flow cytometric analyses. NK, natural killer; NKT, NK-like T; IL, interleukin; WBC, white blood cell; CD3/CD19-depl., CD3/CD19-depleted*.

Following CD3/CD19-depletion 97.8% of all CD45^+^ cells were viable (Figure 3C, Table 2). Median NK cell viability on the day of harvest was 96% following IL-15 stimulation, while viability in both protocols containing an IL-21 boost was slightly lower, but not to a significantly relevant extent (96.2% in the IL-15^low^+IL-21 and 88.3% in the IL-15^gap^+IL-21 protocol). CD3/CD19-depleted cell products consisted in median of 53% NK cells (range 23.0–71.5%) (Figure 3D). By *ex vivo* cultivation for 15 days the NK cell purity could be significantly elevated up to 96.2, 96.5, and 96.7% for IL-15^low^, IL-15^gap^+IL-21, and IL-15^low^+IL-21, respectively. Importantly, there was no significant T or B cell contamination. All NK cell products contained a median amount of 0.01% T cells (range 0–0.04%) and 0.02% B cells (range 0–0.18%) (Table 2). Following CD3/CD19-depletion, the majority of NK cells (94.7%) can be assigned to the CD16^+^ cytotoxic NK cell subpopulation (Figure 3E, Table 2). Upon *ex vivo* cultivation the percentage of CD16^−^ immune regulatory NK cells significantly increased in all three cytokine stimulation protocols up to 49.4% IL-15^low^, 42.7% IL-15^low^+IL-21, and 48.5% IL-15^gap^+IL-21, respectively.

Next, the percentage of lysed target NB cells within the optimized NK cell expansion protocols was evaluated by Europium release assay. Both NB cell lines, SK-N-SH (Figure 4A) and SK-N-AS (Figure 4B), were efficiently lysed by CD3/CD19-depleted NK cells with escalating E:T ratios. The median specific lysis of SK-N-SH cells by IL-15^low^ treated NK cells was 69.5% (range 54.1–82.7%) at an E:T-ratio of 10:1. NK cells further stimulated with IL-21 in the IL-15^low^+IL-21 protocol were able to lyse 71.5% (range 60.0–87.4%) and those cultured in the IL-15^gap^+IL-21 protocol lysed 77.6% (range 70.3–94.3%) of the SK-N-SH cells at an E:T-ratio of 10:1. In summary, the additional IL-21-boost was able to slightly further enhance cytotoxicity. Remarkably, NK cells were even able to lyse SK-N-SH cells at an E:T ratio of 0.5:1 with a median lysis of 8.2% (range 0–16.7%) in the IL-15^low^ protocol, 13.06% (range 6.2–16.1%) following the IL-15^low^+IL-21 treatment and 12.24% (range 10.5–14.7%) in the IL-15^gap^+IL-21 condition (Figure 4A). NK cell mediated killing of SK-N-AS cells was still very effective but slightly lower compared to SK-N-SH cells. NK cells cultured in the IL-15^low^+IL-21 protocol were able to lyse 54.4% (range 39.2–82.2%) and those cultured in the IL-15^gap^+IL-21 protocol 63.3% (range 39.5–76.0%) of SK-N-AS cells at an E:T-ratio of 10:1 (Figure 4B). Overall, NK cells stimulated according to the IL-15^gap^+IL-21 protocol showed the highest cytotoxic potential against both NB cell lines, although differences were not statistically relevant.

### Further Optimization of the *ex vivo* NK Cell Activation Protocol by Specialized NK Cell Medium

To investigate if NK cell expansion can be further enhanced, we included an optimized cell culture medium for the cultivation and expansion of human NK cells, the NK MACS® medium (Miltenyi Biotec) in our stimulation protocols with either continuous IL-15 treatment or with an additional IL-21 boost 2 days prior to cell harvest on day 15 (Figure 5A, Table 3). Comparing the effect of an IL-21 boost to the treatment with IL-15, NK cell expansion was slightly, but not significantly, lower after the boost. Culturing in NK MACS® medium led to a significantly higher expansion rate compared to culturing in X-VIVO^TM^10 medium, which was also used in all previously shown experiments. While NK cells expanded 13.2-fold (range 4–21.6) under IL15 stimulation and 9.6-fold (range 5.1–22.1) after an additional IL-21 boost in X-VIVO^TM^10 medium, expansion rates increased up to 26.4-fold (range 12.7–38.5) and 24.4-fold (range 13.9–41.1), respectively when NK MACS® medium was used (Figure 5B, Table 3).

**Table 3:** Summary statistics to experiments shown in Figure 5.

**Purification method**		**CD3/CD19-depl**.			
**NK cell stimulation**		**IL-15**	**IL-15+IL-21**	**IL-15**	**IL-15+IL-21**
		**X-VIVO10**		**NK MACS**	
NK cell expansion rate	[x-fold]	13.2 (4–21.6)	9.6 (5.1–22.1)	26.4 (12.7–38.5)	24.4 (13.9–41.1)
Viability	[%] of WBC	95.3 (91.6–97.9)	96.4 (90.5–97.3)	98 (97.2–99)	97.3 (96.4–98.2)
Purity NK cells	[%] of WBC	96.1 (95.9–96.4)	96.2 (96–96.6)	96.4 (95.9–96.9)	96.4 (95.6–96.4)
CD16^−^ NK cells	[%] of NK	40.7 (32–51.3)	41.5 (31.9–50)	69.5 (55.7–75.7)	71.6 (58.0–78.9)
CD16^+^ NK cells	[%] of NK	57.3 (43.7–67.1)	57.4 (47–67.4)	29.3 (20.7–42.2)	26.9 (17.5–39.8)
Ratio CD16^−^/CD16^+^		0.7	0.7	2.4	2.7
T cell contamination	[%] of WBC	0.0 (0–0)	0.0 (0–0.04)	0.0 (0–0.01)	0.0 (0–0.01)
NKT cell contamination	[%] of WBC	0.14 (0.11–0.22)	0.21 (0.1–0.25)	0.14 (0.06–0.22)	0.15 (0.13–0.23)
B cell contamination	[%] of WBC	0.06 (0–0.18)	0.14 (0.02–0.18)	0.07 (0.01–0.18)	0.07 (0.02–0.16)

*CD56^+^CD3^−^ NK cells were analyzed on day of harvest (day 10–12). Median (range) expansion rate, purity, viability, NK cell subpopulations and ratios, as well as T, NK-like T and B cell contamination was assessed by flow cytometric analyses. NK, natural killer; NKT, NK-like T; IL, interleukin; WBC, white blood cell; CD3/CD19-depl., CD3/CD19-depleted*.

However, NK cell expansion was not better in NK MACS® compared to that in X-VIVO^TM^10 medium over the entire *ex vivo* cultivation process. Within the first 4 days in culture, NK cell counts decreased and first expansion could be seen in the time period between days 4 and 8. Interestingly, expansion rates during this time point were higher in X-VIVO^TM^10 medium, but after 8 days of culture, NK cell proliferation was significantly better in NK MACS® medium. Further, a continuous increase in expansion could be seen until the day of harvest in NK MACS® medium, while NK cells in X-VIVO^TM^10 medium proliferated until day 15, but with much lower rates than those during the beginning of cultivation (data not shown).

Viability on the day of isolation by CD3/CD19-depletion amounted to 96.7%, which decreased slightly after 15 days of culture in X-VIVO^TM^10 medium. Median viability on the day of harvest of IL-15^low^ treated NK cells was 95.3 and 96.4% after the IL-21 cytokine boost. Culturing NK cells in NK MACS® medium for 15 days increased the median viability up to 98% following IL-15^low^ treatment and up to 97.3% within the IL-15^low^+IL-21 stimulation protocol (Figure 5C, Table 3).

CD3/CD19-depleted cell products consisted prior to expansion (day 0) in median of 44.4% NK cells with T and B cell content of 0.04 and 0.06% (Figure 5D). Fifteen days of *ex vivo* culture led to a high NK cell purity of around 96%, which was independent of cell culture medium and cytokine stimuli (96.1% IL-15^low^ and 96.2% IL-15^low^+ IL-21 after culture in X-VIVO^TM^10 medium and 96.4% IL-15^low^ and 96.4% IL-15^low^+ IL-21 in NK MACS® medium). As observed in the previous results, there was hardly any T or B cell contamination. All NK cell products were almost T and B cell free (range of 0–0.04% T cells and 0–0.18% B cells) (Table 3). Directly after CD3/CD19-depeletion, the predominant NK cell population at 95.9% consisted of CD16^+^ cytotoxic cells (day 0) and decreased to 57.3 and 57.4% under cultivation with IL-15^low^ or IL-15^low^+IL-21 in X-VIVO^TM^10, respectively (day 15, Figure 5E, Table 3). Interestingly, the cultivation in NK MACS® medium led to an even more pronounced effect with significantly higher proportions of CD16^−^ cells, which made up 69.5% of NK cells following culturing with IL-15^low^ and 71.6% after an additional IL-21-boost displayed by a switch in the CD16^−^/CD16^+^ ratio to 2.4 and 2.7, respectively (Figure 5E, Table 3).

### Cytotoxic Potential and Long-Term Cytotoxicity After Optimization of NK Cell Cultivation

The additional IL-21 boost 2 days prior to harvest was able to significantly enhance cytotoxicity compared to cultivation with only IL-15^low^ (Figure 6A). This observation was seen in both NK cell media but was even more pronounced for NK cells cultivated in NK MACS® medium. Here, even at an E:T ratio of 0.5:1, the addition of IL-21 led to an significant increase in target cell lysis. However, specific cell lysis was slightly higher after *ex vivo* culture in X-VIVO^TM^10 medium compared to that in NK MACS® medium. At an E:T ratio of 10:1, the specific lysis of SK-N-SH cells by IL-15^low^-treated NK cells in X-VIVO^TM^10 medium was 73.6% (range 59.2–97.8%). An IL-21 boost elevated lysis up to 94.9% (range 66.3–100%). In NK MACS® medium, lysis was 72.1% (range 64.6–81.1%) for IL-15^low^ and 80.1% (range 68.1–89.2%) for IL-15^low^+IL-21 (Figure 6A). At an E:T ratio of 10:1, the specific lysis of SK-N-AS cells by IL-15^low^ stimulated NK cells in X-VIVO^TM^10 medium was 57.4% (range 46.9–86.3%), and an IL-21 boost elevated the lysis up to 68.1% (range 57.4–92.4%). In NK MACS® medium, lysis was 50.7% (range 36.0–59.8%) (IL-15^low^) and 57.8% (range 45.6–78.0% (IL-15^low^+ IL-21) (Figure 6B). Remarkably, NK cells did not only lyse NB cells at high E:T ratios but also at very low ones. Median specific lysis of SK-N-SH cells at an E:T of only 0.5:1 was 8.7% (range 3.5–13.3%) for IL-15^low^ X-VIVO^TM^10, 9.2% (range 1.4–16.4%) for IL-15^low^+IL-21 X-VIVO^TM^10, 5.6% (range 0–9.0%) for IL-15^low^ NK MACS® and 9.6% (range 7.7–13.0%) for IL-15^low^+IL-21 in NK MACS® medium.

To further address long-term cytotoxicity, NB tumor spheroids built of SK-N-AS cells were co-incubated with IL-15^low^+IL-21 stimulated NK cells cultured in either X-VIVO^TM^10 NK or MACS® medium. As already demonstrated in the short-term cytotoxicity assays, no differences between both media were observed in long-term cytotoxicity assays using tumor spheroids. Regardless what culture medium was used, IL-15^low^+IL-21 stimulated NK cells were able to quickly infiltrate and completely lyse the tumor spheroids (Figure 6C).

### IFN-γ Production and Expression of the Degranulation Marker CD107a

Degranulation assays detecting CD107a as a marker for stimulation-induced granule exocytosis and intracellular IFN-γ staining were performed comparing NK cells previously cultured with IL-15^low^ or IL-15^low^+IL-21 in X-VIVO™10 or NK MACS® media. As degranulation/cytokine production stimulus either SK-N-AS NB target cells (E:T ratio 1:1) or IL-12+IL-18, mimicking stimulation by dendritic cells, was used (Figure 7). Both NK cell subpopulations were capable of IFN-γ production upon cytokine stimulation and target cell co-incubation and higher levels of IFN-γ^+^ cells were detected following cytokine stimulus compared to tumor cell co-incubation, which was statistically significant for the CD16^−^ subset. Between both culture media similar effects were observed, except CD16^−^ NK cells grown in X-VIVO™10 produced significantly more IFN-γ upon IL-12+IL-18 cytokine stimulation. Target cell co-incubation and cytokine stimulation led to a high CD107a expression in the different NK cell subpopulations, thereby more pronounced within the CD16^−^ NK cell population. Only small differences were seen between both cell culture media, but throughout all experiments, the additional IL-21 boost during NK cell cultivation enhanced IFN-γ and CD107a expression, which was noticeable in all measurements and was even statistically significant in *n* = 2 settings.

### Phenotype Analysis of NK Cells and CD16^−^ and CD16^+^ Subpopulations

**Figure 8:**
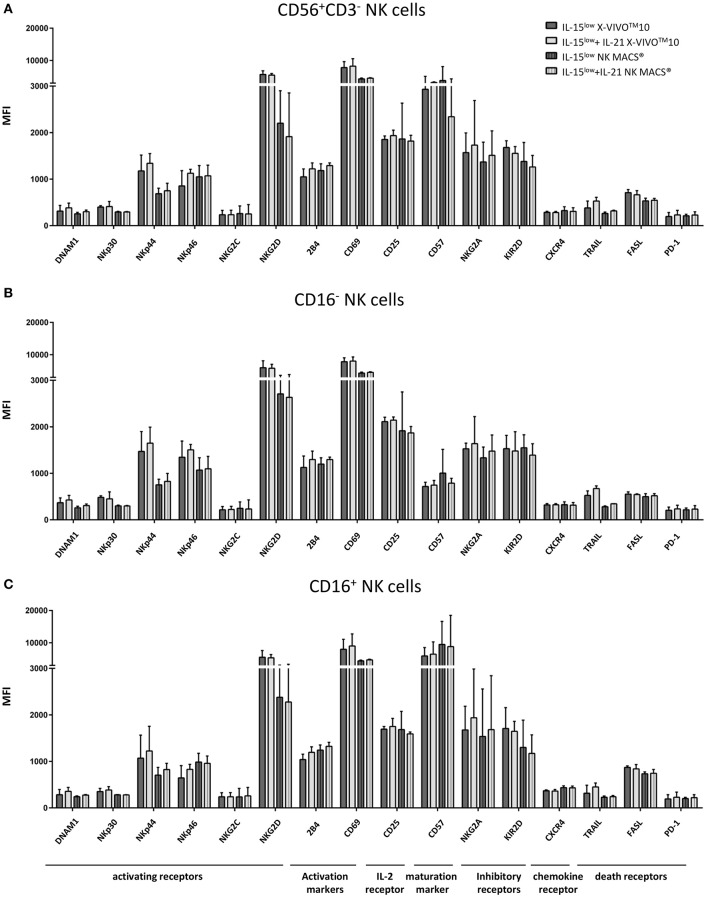
Phenotype analyses of NK cells and CD16^−^ and CD16^+^ subpopulations. **(A)** Expression of various surface markers on NK cells including CD16^−^ **(B)** and CD16^+^ **(C)** NK cell subpopulations on the day of harvest. No significant differences between stimulation with IL-15 solely (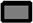

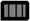
) or in combination with IL-21 (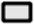

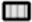
) could be seen. The activating receptors NKp44 and NKG2D and the activation marker CD69 showed higher expression on NK cells cultured in X-VIVO^TM^10 medium (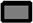

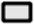
) compared to ones cultured in NK MACS® medium (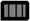

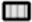
). While the CD16^+^ NK cell population expressed higher levels of maturation marker CD57, the inhibitory receptor NKG2A and the death receptor FASL, CD16^−^ NK cells expressed the activating receptors NKp44 and NKp46 as well as the α-chain of the IL-2/IL-15 receptor CD25 to a higher extend (statistically not relevant differences). *n* = 4, independent results, median fluorescence intensity (MFI), bar graphs show median and interquartile range, gated on viable 7-AAD^−^ NK cells using FMO (fluorescence minus one) controls for each antigen.

Extensive flow cytometric analysis revealed that the IL-21 boost had no significant influence on the expression of a broad repertoire of NK cell characteristic cell surface markers when compared to stimulation with IL-15^low^ only (Figure 8A). Notably, the activating cytotoxicity receptors NKp44 and NKG2D and the activation marker CD69 showed higher expression on NK cells following cultivation in X-VIVO^TM^10 medium compared to NK MACS® medium. However, all other surface markers tested were equally expressed on NK cells cultured in either X-VIVO^TM^10 or NK MACS® medium.

Irrespective of culture medium-dependent differences, also variations in the receptor expression of CD16^−^ and CD16^+^ NK cell subpopulations were observed (Figures 8B,C). Receptors associated with cytotoxicity, particularly NKG2D which plays a crucial role in NK cell killing, were equally expressed on both NK cells subsets following expansion by IL-15^low^ and IL-15^low^+IL-21; NKp44 and NKp46 even to a higher extend in CD16^−^ NK cells. In the same line, expression of CD25, the α-chain of the IL-2/IL-15 receptor, seemed to be correlated to activation. By contrast, the death receptor FASL and the maturation marker CD57 were considerably higher expressed on CD16^+^ NK cells, which was expected as the CD56^dim^CD16^+^ subset is described as the mature, while CD16^−^ NK cells are the more immature NK cell subpopulation.

## Discussion

Current therapy for high-risk NB includes surgery, myeloablative conditioning and autologous SCT, but the occurrence of relapse remains still a major limit (31, 32). Despite therapeutic improvements, the overall survival is not satisfying, and alternative treatment options are urgently needed. To benefit from the GvL/T effect, allogeneic SCT was considered for NB patients. However, there was no difference in the outcome of patients treated with autologous purged and matched allogeneic SCT (33). Haploidentical SCT is an option to enhance the GvL/T effect but carries an increased risk of severe GvHD, mainly mediated by alloreactive donor T cells. An effective strategy to prevent the occurrence of lethal GvHD is the *in vitro* depletion of T cells. Specific depletion techniques are available to remove T and B cells from the grafts (CD3/CD19- or TCRαβ/CD19-depletion) while preserving cells that are beneficial regarding engraftment and GvL/T effect such as NK cells (9, 34, 35). Notably, HLA class I expression is reduced on NB cells which makes them valid targets for NK cell lysis (10, 36). The feasibility of haploidentical SCT for solid tumors including NB was shown, unfortunately with limited effect (6, 8, 37–39). Most studies included small cohorts of patients who suffered from diverse primary tumors (6, 38) that relapsed following autologous SCT (6) or were in non- or partial remission at the time-point of haploidentical SCT (6, 8, 38, 39). Illhardt et al. treated 26 patients suffering from refractory or relapsed metastatic NB showing that patients reaching complete remission before haploidentical SCT had a significantly better prognosis compared to patients with proven residual tumor load. Unfortunately, event-free and overall-survival in the whole cohort at 5 years post SCT were 19% and 23%, respectively, enforcing the urgent need for improved treatment options (8).

Adoptive immunotherapy following SCT is a promising approach to further strengthen GvL/T to combat relapse in high-risk solid tumors, such as NB. Within the last decade, different cell-based immunotherapeutic strategies were adopted and refined, including the infusion of donor lymphocytes (DLI), NK cells with and without prior cytokine stimulation, and CIK cells (18, 20, 40–42). CIK cells have shown high anti-leukemic potential, while demonstrating only low alloreactive potential. Through their heterogeneous composition, including a majority of CD3^+^ T cells, CD3^+^CD56^+^ NK-like T cells and a small amount of CD3^−^CD56^+^ NK cells, CIK cells can mediate different killing mechanisms involving T cell receptor-dependent and non-HLA-restricted cytotoxicity using various receptors and signaling pathways (27, 40, 43, 44). In a previous work, we showed that CIK cells were able to attack NB by lysing between 14.7 and 19.8% of different NB cell lines, such as UKF-NB-3, UKF-NB4 and SK-N-SH, at an E:T ratio of 20:1 (27). Hence, repeated CIK cell immunotherapy might support the patient's immune system to combat NB relapse, but increased killing rates are desirable. An encouraging approach might be the engineering of CAR-CIK cells as already shown for refractory or advanced soft tissue sarcoma (28).

Another immunotherapeutic strategy that was successfully applied in NB patients is the infusion of allogeneic NK cells (18, 19, 45). The expression of stress-induced activating receptor ligands MHC class I chain-related protein A and B (MICA/B) combined with reduced expression of HLA class I surface molecules to escape T cell mediated killing, renders NB an optimal target for NK cell mediated cytotoxicity (46). Within clinical trials, we and others applied highly purified NK cells with and without prior *ex vivo* IL-2 stimulation (19, 20, 47). Thereby, we have demonstrated feasible manufacturing under GMP conditions and the safe application of haploidentical NK cells on day +40 and +100 post SCT in 16 pediatric patients suffering from high-risk malignancies (4 of them with high-risk NB stadium IV). Two of the four patients with NB treated with IL-2-stimulated NK cells showed a notable response. One patient is still alive 9.3 years after SCT and NK cell immunotherapy whereas the second patient remained in complete remission (CR) for 5.8 years. However, despite a high lytic activity of the activated donor NK cells *in vitro* and these promising clinical responses, the continuation of this clinical study was limited by an NK cell expansion rate of a median 4-fold, which did not ensure repetitive high-dose NK cell infusions. In addition, the manufacturing procedure was associated with enormously high expenses.

In substantial research studies, various cytokine combinations with and without feeder cell addition were tested to investigate the improvement of NK cell proliferation and cytotoxicity against a broad range of tumor entities (23, 48, 49). The establishment of a protocol without feeder cells seems advantageous as cell products produced with feeder cells cannot simply be infused, but must be thoroughly tested for clinical safety first (50–52). In this work, we generated NK cells with IL-2, IL-15 and/or IL-21 using either CD3/CD19-depleted or CD56-enriched PBMCs as starting material. Thereby, our analyses revealed that NK cells generated from CD3/CD19-depleted starting material expanded significantly faster compared to conventionally generated CD56-enriched NK cells. This might be due to autologous accessory cells of the CD3/CD19-depleted cell product, meaning co-cultured non-NK cells, such as monocytes and DCs, that can facilitate NK cell expansion and stimulation by cytokine secretion and/or direct cell-to-cell-interaction (53–55). Monocytes analyzed early in co-culture of CD3/CD19-depleted NK cell products expressed the high affinity IL-15 receptor alpha (IL-15Rα, CD215) on the surface, indicating a possible mechanism of trans-presentation of IL-15 to NK cells by the IL-2Rβ and γ chain receptor complex. Similar to the recently described design of a IL-15 superagonist by complexing IL-15 and IL-15Rα in solution, this may as well enhance the biological activity of IL-15 in the expansion procedure of NK cells when co-cultured with accessory cells such as monocytes following CD3/CD19-depletion. This effect might explain the enhanced NK cell proliferation of CD3/CD19-depleted PBMCs compared to highly purified CD56-enriched NK cell cultures (56–58). Unlike to classical feeder cells, these accessory cells are not of third-party origin and do not need to be inactivated or irradiated. With regard to an intended clinical cell application, this is of crucial importance, because these cells are not going to be removed prior to clinical cell application. Moreover, in the clinical haploidentical setting that carries an elevated risk of developing GvHD, the amount and possible expansion of residual T cells needs to be carefully monitored. Importantly, the NK cell product generated from CD3/CD19-depleted primary material consisted of only 39% NK cells at culture start, but reached a purity of almost 99%, without relevant T or B cell contamination (<0.2%).

We further showed that NK cells isolated by CD3/CD19-depletion exerted significantly higher cytotoxic activity than those isolated by CD56-enrichment. These findings are coherent to findings published by Williams et al. (59). In addition to the common 2D cytotoxicity tests, we performed lysis experiments with tumor spheroids. Hereby, using the Celigo cell cytometer, we were able to investigate killing over an extended time period up to 10 days and in a 3D structure closer to *in vivo* tumor conditions (60–62). All NK cells were able to suppress tumor growth and the superior lysis of CD3/CD19-depleted cells could be affirmed. However, especially NK cells generated from CD3/CD19-depleted PBMCs cultured with IL-15 mediated remarkably high cytotoxicity in short-term and long-term killing of the 3D spheroid structure without reoccurrence of target cells. Interestingly, while CIK cells showed significantly lower short-term killing capacity, in the long-term cytotoxicity assay against 3D NB structures, CIK cells eradicated tumor cells as well. This was probably due to their persistence and proliferative capacity under these specific *in vitro* conditions. Furthermore, CIK-cell-mediated killing is a process that is known to need more time than NK cell-induced killing (63).

Although IL-2-stimulated NK cells were successfully administered in several clinical studies, we and others showed advantageous expansion rates and cytotoxicity with IL-15 stimulation (50, 64). IL-2 is described as a relevant cytokine for the maintenance of induced regulatory T cells and also for the activation and proliferation of T cells which might be of importance regarding tumor-escape mechanisms, but also for GvHD development (65–67).

Therefore, encouraging results were published by Choi et al. treating 41 adult patients suffering mainly from leukemia with haploidentical NK cell immunotherapy generated from CD3-depleted primary material stimulated with IL-15, IL-21 and hydrocortisone over a period of 13 to 20 days. The median cell dose given in this study was with 2 × 10^8^ NK cells kg/BW ~10–20 times higher compared to previous studies leading to a significant reduction in leukemia progression (68). Experience with NK cell expansion from CD3/CD19-depleted primary material was also published by Williams et al. and van Ostaijen-ten Dam et al., however their cultivation with IL-2 and/or IL-15 was limited to overnight and 5 days, resulting in lower cell expansion compared to our protocol (59, 69).

Here, we could see that NK cells isolated by CD56-enrichment and cultured with IL-2 in addition to IL-15 showed higher T cell contamination (median 1%) than NK cells cultured with IL-15 only. Stimulation with IL-21 is known to enhance NK cell cytotoxicity, while hampering IL-2- and IL-15-driven expansion and viability (50, 70–72). As already shown by Wagner et al. (23), an additional short-term IL-21 boost to IL-15^low^-cultured NK cells prior to harvest further significantly increased cytotoxicity and partly also NK cell expansion. They described, that with the IL-15+IL-21 boost protocol, expansion rates ranged between 2- and 10-fold depending on the donor, exhibiting an average 4.5-fold increase on day 10 of culture. In our experiments, the effect of the IL-21 boost on NK cell expansion was also to some extend variable and donor-dependent, while IL-21 consistently increased NK cell cytotoxicity, in median 8–10% when NK cells were cultured in the NK cell optimized medium NK MACS®. Remarkably, this gain in cytotoxicity was even more significant than described by Wagner et al. (23).

Importantly, our data highlight that cultivation of NK cells from CD3/CD19-depleted primary material in NK MACS® medium compared to X-VIVO^TM^10 medium resulted in significantly higher expansion rates up to 26.4-fold compared to 13.2-fold following IL-15^low^ stimulation, which was combined with higher purity and viability, but slightly lower cytotoxicity against NB cell lines. Oberschmidt et al. reported a median expansion of 5.9-fold after 14 days of *ex vivo* cultivation of NK cells in NK MACS® medium with the addition of IL-2/IL-15 and initial IL-21 after CD3-depletion and CD56-selection from leukapheresis products using the CliniMACS® Prodigy device (73). Significantly higher expansion rates using NK MACS® compared to X-VIVO^TM^10 media were also described by Klöß et al. when cultivating NK cells (74).

In our study NK cell subsets were analyzed for the distribution of the CD56^bright^CD16^−^ and CD56^dim^CD16^+^ subpopulation. As all stimulation protocols induced an upregulation of the NK cell marker CD56, leading to a complete CD56^bright^ phenotype, only a discrimination of CD16^+^ and CD16^−^ NK cells was used to define distinct subpopulations in the resulting cell products. Upon cytokine stimulation we further observed an increase of the CD16^−^ NK cell population. This was in correlation with a previous report describing a reduction of CD16^+^ NK cells and an increase of CD16^−^ NK cells upon IL-15 and IL-15+IL-21 simulation (23). It has been shown earlier that CD16^−^ NK cells have a high proliferating capacity. Klingemann et al. reported that *ex vivo* culture of CD56 cells leads primarily to expansion of the CD16^−^ cell population, when stimulated with IL-2 or IL-2+IL-15 in X-VIVO™10 media for 14 days (75). Interestingly, our data showed that the increase of the CD16^−^ proportion was more pronounced when cells where generated by CD3/CD19-depletion and even led to an inverse distribution in the proportion of CD16^−^ and CD16^+^ NK cell subsets when cultured in NK MACS® compared to X-VIVO™10 medium (see Tables 1–3). This outgrowth of the CD16^−^ subpopulation correlated with a significantly increased expansion capacity when NK cells were cultured in NK MACS® medium, indicating that CD16^−^ NK cells are the major subpopulation stimulated under these given cell culture conditions.

When addressing the impact of different culture conditions on the NK cell proliferation capacity, the culture in the presence of NK MACS® led to the highest expansion rates, while expansion was significantly lower in the other tested media X-VIVO^TM^10. This observation is in line with a report by Klöß et al. on a detailed comparison of different GMP-compliant expansion media. In addition, they also describe an altered proportion of CD16^−^ and CD16^+^ NK cell subsets in all culture batches containing NK MACS® medium, with in median 49.6% (range 42.2–51.5) CD16^dim/−^ and 50.4% (range 48.6–57.2) CD16^+^ containing NK cells (74). These findings are also consistent with a study describing a wide range of CD16^−^/CD16^+^ ratio between 0.2 and 2.3 (median 0.9) when CD3-depleted CD56-enriched NK cells were cultured in NK MACS® medium + IL-2/IL-15 and initial IL-21 for 14 days using the CliniMACS Prodigy device (73).

Regarding a future clinical application of NK cells stimulated by IL-15^low^+IL-21 in NK MACS® for 15 days, the characterization of the final NK cell phenotype and function is of crucial interest. Importantly, cytotoxic activity did not differ between both cell culture medium conditions, as we have demonstrated in short- and long-term cytotoxicity experiments, thereby indicating that the CD16^−^ population also exerts potent killing mechanisms. Moreover, both NK cell subsets were capable of IFN-γ production as well as CD107a expression. Contact with NB tumor cells or cytokine stimulation with IL-12+IL-18, mimicking activation by dendritic cells, led both to a CD107a expression in both NK cell subpopulations, and was even more pronounced within the CD16^−^ NK cell population. Interestingly, in correlation to the natural NK cell function *in vivo*, the cytokine stimulus induced a high cytokine (IFN-γ) production in both NK cell subsets, while NK cells responded to the NB cell co-incubation with enhanced CD107a expression. Notably, especially the CD16^−^ NK cell population showed poly-functional characteristics being capable of both cytokine secretion and degranulation.

These findings are in line with recent publications investigating the systemic IL-15 administration in cancer patients. Dubois et al. describe a dramatic expansion with a 358-fold increase of CD56^bright^ NK cells exceeding the CD56^dim^ NK cell populations upon continuous i. v. IL-15 infusions. As expected, CD56^bright^ NK cells responded with increased cytokine release to various stimuli. Importantly, CD56^bright^ NK cells also gained the ability to kill various target cells. In addition, IL-15 treatment resulted in increased amounts of the cytolytic molecules granzymes A and B and perforin in CD56^bright^ NK cells, what conclusively led the authors to state that IL-15 infusions appear to equip CD56^bright^ NK cells to become cytotoxic and therefore enabled the whole NK cell population to mediate antitumor response (76, 77). Our results are also consistent with recent findings describing CD56^bright^ NK cells exhibiting potent antitumor responses with multiple cell functions such as degranulation, cytotoxicity and cytokine production following IL-15 priming (78). Wagner et al. postulate that IL-15 unleashes potent antitumor effector function of CD56^bright^ NK cells. Traditionally, CD56^bright^CD16^−^ NK cells are described as cells with low antitumor responsiveness that are primarily involved in immune regulatory functions like cytokine secretion. However, these studies in correlation to our report indicate that this definition may not be transferable to *in vitro* cytokine stimulated NK cells.

In addition, also receptors associated with cytotoxicity, i.e., DNAM1, NKp30 and particularly NKG2D which plays a crucial role in NK cell killing, were equally expressed in both NK cell subsets after IL-15^low^ and IL-15^low^+IL-21 treatment. NKp44 and NKp46 even to a higher extend in CD16^−^ NK cells, underlining the gain in cytotoxic potential of this subset. In our study, we observed that an additional IL-21 boost to continuous IL-15^low^ treatment significantly enhanced NK cell cytotoxicity, which was even more pronounced when NK cells were cultured in NK MACS® medium. However, also in accordance with our previous reports, surface expression of the corresponding receptors did not correlate with these findings (23). Klöß et al. found no differences in the expression of the markers NKp30, NKp44, NKp46, and NKG2D between NK cells cultured in X-VIVO^TM^10 and NK MACS® medium (74), while Oberschmidt et al. reported the upregulation of NKG2D, NKp30, and NKp44 on NK cells cultured for 14 days in NK MACS® medium with IL-2/IL-15 and initial IL-21 relative to unstimulated cells (73).

Overall, in our study we address whether donor NK cells may provide a potent cell-based immunotherapeutic approach to treat high-risk NB patients in addition to haploidentical SCT. We report on our efforts to optimize the cytokine stimulation protocol with regard to expansion rate and cytotoxic activity of NK cells generated from CD3/CD19-depleted products in comparison to CD56-enriched NK cells or CIK cells activated by our well-established manufacturing procedure. In sum, our data revealed that NK cells have a significantly higher cytotoxic potential to combat NB than CIK cell products. Although CIK cells have shown compelling results in the treatment of hematological malignancies, NK cells seem superior regarding the targeting of NB.

As an important step toward clinical application, these results could be verified in a first *proof of principle experiment* in which we analyzed NK cells from a clinical CD3/CD19-depleted G-CSF stimulated stem cell leukapheresis product using the IL-15^low^+IL-21 stimulation protocol. Expansion rate, viability, and cytotoxicity against NB target cells were similar to NK cells isolated from PBMCs. On the base of our finding, for subsequent studies we favor the use of NK cells generated from CD3/CD19-depleted cell products stimulated in a 15 day expansion procedure using IL-15^low^+IL-21 and NK MACS medium. Up-scaling experiments toward the translation into a GMP-compliant expansion protocol are ongoing, as well as the establishment of a Xenograft mouse model to verify our data *in vivo*.

As one major limitation of repeated NK cell immunotherapy is the generation of sufficient amounts of NK cells to apply clinically relevant doses, this optimized expansion and stimulation protocol to *ex vivo* cultivate NK cells from grafts of a haploidentical donors might be used in future clinical studies. In this context, the use of IL-15- and IL-21-expanded NK cells generated from CD3/CD19-depleted apheresis products seem to be highly promising as additional immunotherapy in combination with haploidentical SCT for high-risk NB patients.

## Data Availability Statement

The datasets generated for this study are available on request to the corresponding author.

## Ethics Statement

The studies involving human participants were reviewed and approved by Ethics Committee of the Goethe University Frankfurt, Germany (approval no. 329/10). The patients/participants provided their written informed consent to participate in this study.

## Author Contributions

AH, CC, and EU conceived and designed the experiments. AH, BG, JF, TM, and LG performed the experiments. AH, BG, CC, and SH analyzed the data. EU, PB, and TK coordinated and supervised the research. CC, EU, ER, JF, SH, MM, and JS discussed the results. AH, BG, CC, EU, and MB wrote the paper with support from all co-authors. All authors approved the final version of the manuscript.

### Conflict of Interest

The authors declare that the research was conducted in the absence of any commercial or financial relationships that could be construed as a potential conflict of interest.

## Supplementary Material

Supplementary Figure 1 Characterization of CIK and NK cells supplemental to Figure 1. **(A)** CD56-enriched NK cell products contained a median of 1.0 and 0.3% T cells and CD3/CD19-depleted NK cell products a median of 0.03% and 0.05% T cells following IL-2+IL-15 and IL-15 stimulation, respectively. T cell contamination was significantly higher in CD56-enriched NK cell products and if IL-2 was added. Frequency of T cells within the CIK cell bulk increased following cytokine stimulation from 54.7% up to 84.2%. **(B)** CD56-enriched NK cell products contained a median of 0.5% and 0.16% NK-like T cells and CD3/CD19-depleted NK cell products in median 0.08% and 0.03% NK-like T cells following IL-2+IL-15 and IL-15 stimulation, respectively. Frequency of NK-like T cells within the CIK cell bulk increased following cytokine stimulation from 3.7% up to 6.2%. **(C)** CD56-enriched NK cell products contained a median 0.04% and 0.08% B cells and CD3/CD19-depleted NK cell products contained a median 0.1% and 0.1% B cells following IL-2+IL-15 and IL-15 stimulation, respectively. Frequency of B cells within the CIK cell bulk decreased following cytokine stimulation from 6.5% on day 0 to 0.06% (*n* = 12 independent experiments, median fold expansion rate day 10–12 compared to day 0), gated on viable cells (DAPI negative): CD3^+^ T cells **(A)**, CD3^+^CD56^+^ NK-like T cells **(B)**, and CD19^+^ B cells **(C)**. Differences were considered significant for *p* < 0.05 (^*^), *p* < 0.01 (^**^), and *p* < 0.001 (^***^).
