# Supplementary figures and images for: The Synergistic Use of IL-15 and IL-21 for the Generation of NK Cells From CD3/CD19-Depleted Grafts Improves Their ex vivo Expansion and Cytotoxic Potential Against Neuroblastoma: Perspective for Optimized Immunotherapy Post Haploidentical Stem Cell Transplantation
Source: Front Immunol. 2019 Dec 3;10:2816. doi: 10.3389/fimmu.2019.02816 (PMC6901699; doi:10.3389/fimmu.2019.02816)

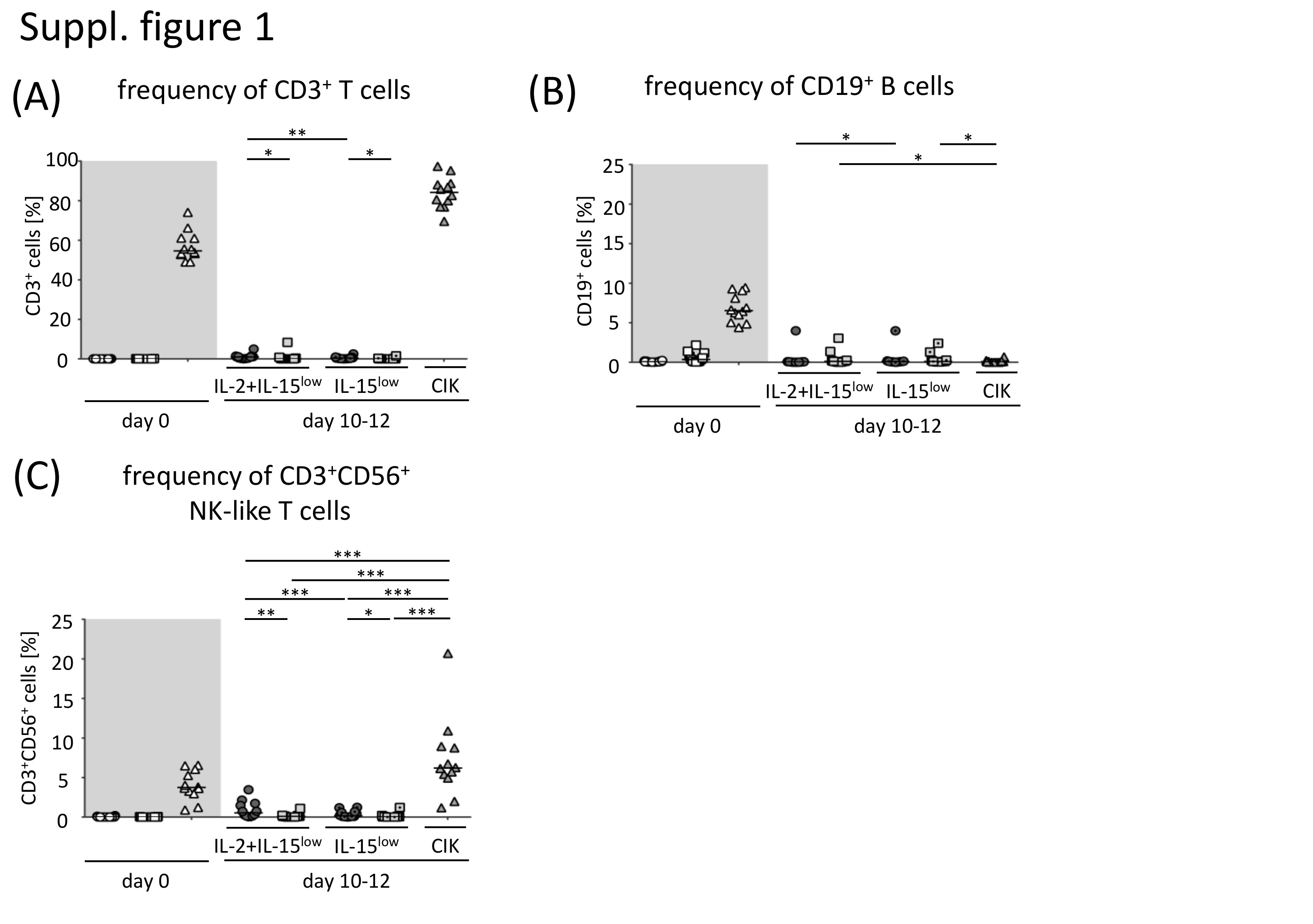

Supplement: Supplementary Figure 1 — Characterization of CIK and NK cells supplemental to Figure 1. (A) CD56-enriched NK cell products contained a median of 1.0 and 0.3% T cells and CD3/CD19-depleted NK cell products a median of 0.03% and 0.05% T cells following IL-2+IL-15 and IL-15 stimulation, respectively. T cell contamination was significantly higher in CD56-enriched NK cell products and if IL-2 was added. Frequency of T cells within the CIK cell bulk increased following cytokine stimulation from 54.7% up to 84.2%. (B) CD56-enriched NK cell products contained a median of 0.5% and 0.16% NK-like T cells and CD3/CD19-depleted NK cell products in median 0.08% and 0.03% NK-like T cells following IL-2+IL-15 and IL-15 stimulation, respectively. Frequency of NK-like T cells within the CIK cell bulk increased following cytokine stimulation from 3.7% up to 6.2%. (C) CD56-enriched NK cell products contained a median 0.04% and 0.08% B cells and CD3/CD19-depleted NK cell products contained a median 0.1% and 0.1% B cells following IL-2+IL-15 and IL-15 stimulation, respectively. Frequency of B cells within the CIK cell bulk decreased following cytokine stimulation from 6.5% on day 0 to 0.06% (n = 12 independent experiments, median fold expansion rate day 10–12 compared to day 0), gated on viable cells (DAPI negative): CD3+ T cells (A), CD3+CD56+ NK-like T cells (B), and CD19+ B cells (C). Differences were considered significant for p < 0.05 (*), p < 0.01 (**), and p < 0.001 (***). [file Image_1.JPEG]
